# Advancing presurgical non-invasive spread through air spaces prediction in clinical stage IA lung adenocarcinoma using artificial intelligence and CT signatures

**DOI:** 10.3389/fsurg.2024.1511024

**Published:** 2025-01-14

**Authors:** Guanchao Ye, Guangyao Wu, Yiying Li, Chi Zhang, Lili Qin, Jianlin Wu, Jun Fan, Yu Qi, Fan Yang, Yongde Liao

**Affiliations:** ^1^ Department of Thoracic Surgery, Union Hospital, Tongji Medical College, Huazhong University of Science and Technology, Wuhan, China; ^2^ Department of Radiology, Union Hospital, Tongji Medical College, Huazhong University of Science and Technology, Wuhan, China; ^3^ Department of Breast Surgery, The First Affiliated Hospital of Zhengzhou University, Zhengzhou, China; ^4^ Department of Radiology, Dalian Public Health Clinical Center, Dalian, China; ^5^ Department of Radiology, The Affiliated Zhongshan Hospital of Dalian University, Dalian, China; ^6^ Department of Pathology, Union Hospital, Tongji Medical College, Huazhong University of Science and Technology, Wuhan, China; ^7^ Department of Thoracic Surgery, The First Affiliated Hospital of Zhengzhou University, Zhengzhou, China

**Keywords:** spread through air spaces, lung adenocarcinoma, radiomics, surgical strategy, artificial intelligence

## Abstract

**Background:**

To accurately identify spread through air spaces (STAS) in clinical stage IA lung adenocarcinoma, our study developed a non-invasive and interpretable biomarker combining clinical and radiomics features using preoperative CT.

**Methods:**

The study included a cohort of 1,325 lung adenocarcinoma patients from three centers, which was divided into four groups: a training cohort (*n* = 930), a testing cohort (*n* = 238), an external validation 1 cohort (*n* = 93), and 2 cohort (*n* = 64). We collected clinical characteristics and semantic features, and extracted radiomics features. We utilized the LightGBM algorithm to construct prediction models using the selected features. Quantifying the contribution of radiomics features of CT to prediction model using Shapley additive explanations (SHAP) method. The models' performance was evaluated using metrics such as the area under the receiver operating characteristic curve (AUC), negative predictive value (NPV), positive predictive value (PPV), sensitivity, specificity, calibration curve, and decision curve analysis (DCA).

**Results:**

In the training cohort, the clinical model achieved an AUC value of 0.775, the radiomics model achieved an AUC value of 0.836, and the combined model achieved an AUC value of 0.837. In the testing cohort, the AUC values of the models were 0.743, 0.755, and 0.768. In the external validation 1 cohort, the AUC values of the models were 0.717, 0.758, and 0.765, while in the external validation 2 cohort, 0.725, 0.726 and 0.746. The DeLong test results indicated that the combined model outperformed the clinical model (*p* < 0.05). DCA indicated that the models provided a net benefit in predicting STAS. The SHAP algorithm explains the contribution of each feature in the model, visually demonstrating the impact of each feature on the model's decisions.

**Conclusion:**

The combined model has the potential to serve as a biomarker for predicting STAS using preoperative CT scans, determining the appropriate surgical strategy, and guiding the extent of resection.

## Introduction

In 2015, Kadota introduced the concept of spread through air spaces (STAS), which is characterized by the presence of tumor cells in the air spaces of lung parenchyma beyond the main tumor mass (1). The World Health Organization integrated the concept of STAS into the novel invasion model for lung adenocarcinoma, categorizing its pathological features into three types: micropapillary cluster, solid tumor nest, and single free tumor cell (2). Currently, the clinical significance of STAS has garnered increasing attention. Numerous studies have investigated the association between STAS and the clinicopathological characteristics and semantic features of lung cancer, as well as its prognostic implications for patients with early-stage lung cancer treated with various surgical approaches (3, 4). An increasing body of evidence demonstrated that the presence of STAS substantially diminishes the overall survival (OS) and recurrence-free survival (RFS) rates in lung cancer, particularly in stage I lung adenocarcinoma (5–7).

Furthermore, due to the improved detection of small peripheral lung cancer facilitated by the widespread application of CT, sub-lobar resection (including wedge resection and segmental resection) has gained popularity for the management of clinical stage IA lung cancer (8, 9). However, STAS is linked to locoregional recurrence in patients undergoing sub-lobar resection for lung cancer (10). Studies have reported a heightened risk of distant and local recurrence following sub-lobar resection of STAS-positive tumors, a risk not observed in patients undergoing lobectomy (1, 3). Hence, preoperative identification of STAS aids in selecting the most suitable surgical approach.

In clinical practice, the most frequently employed techniques for intraoperative or preoperative diagnosis of STAS are chest CT scan, biopsy, and intraoperative frozen section (FS) analysis (11). Preoperative CT scans facilitate non-invasive diagnosis of STAS, assisting in the selection of a tailored surgical approach. Numerous radiological studies rely on morphological (semantic) features, including size, presence of solid components, spiculation, or lobulation, to diagnose STAS (12, 13). However, the qualitative interpretation of images is impeded by the subjective nature of atypical radiological signs. Biopsy is an invasive examination that further increases the possibility of tumor cell dissemination. The sensitivity of intraoperative frozen identification of STAS needs to be improved.

Radiomics provides a non-invasive method to capture additional information that cannot be seen by the naked eye by extracting high-throughput image features from a large number of medical images and performing relevant analysis on these advanced features (14). Based on the existing literature, a substantial number of studies have investigated the relationship between radiomics features and STAS, integrating these features into machine learning algorithm to develop predictive models for STAS (15–18). However, the non-interpretability of radiomics models hinders their widespread use. The Shapley additive explanations (SHAP) algorithm is currently the most recommended algorithm for model interpretation, as it can explain how the values of each feature affect the effects attributed to the model's features and integrate the effects of features attributed to individual responses through visualization. Therefore, we investigate the use of SHAP algorithm to visualize and interpret the STAS model based on its construction, in order to understand the contribution of each feature to the model's decision-making. In addition, the current study examined a cohort of 1,325 patients diagnosed with clinical stage IA lung adenocarcinoma from three institutions, rendering it the most extensive investigation thus far in terms of the number of centers and cases.

Radiomics features were extracted from preoperative CT scans and integrated with clinical characteristics through machine learning algorithms to develop a predictive biomarker for STAS. We use SHAP algorithm to further understand the internal mechanism of the model, which has better interpretability and facilitates clinical communication and interpretation. This non-invasive and interpretable biomarker supports clinicians in devising tailored treatment strategies and choosing personalized surgical approaches, aided by artificial intelligence.

## Materials and methods

### Study population

This retrospective study, registered at http://clinicaltrials.gov (identifier: NCT05400304), obtained approval from the institutional review boards and the Ethics Committee of Union Hospital, Tongji Medical College, Huazhong University of Science and Technology (identifier: UHCT22749). Informed consent requirements were waived.

**Figure 1:**
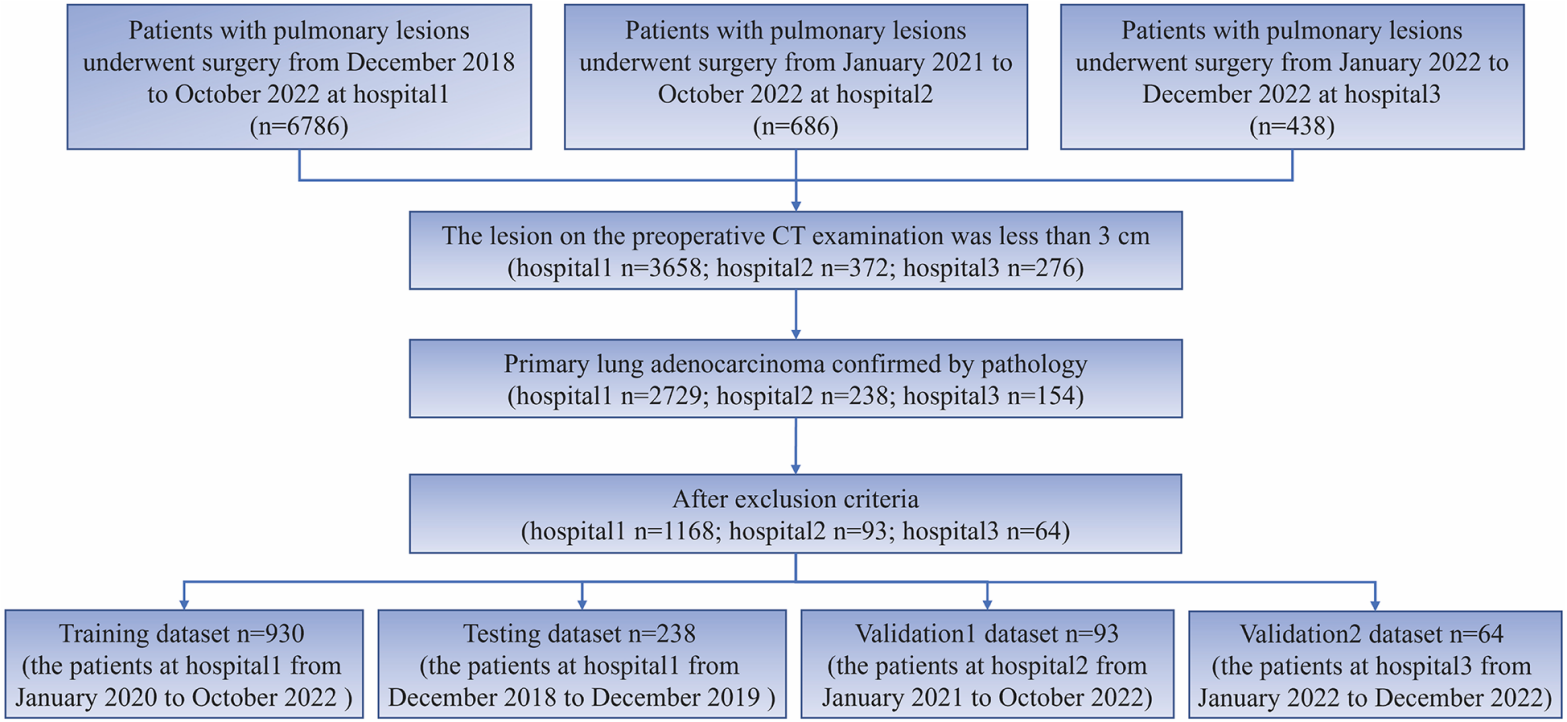
Flowchart for patients’ selection from three hospitals.

This study employed the following inclusion criteria: (1) preoperative CT examination indicating a lesion smaller than 3 cm, and (2) Patients with postoperative pathological confirmation of lung adenocarcinoma. The following exclusion criteria were applied: (1) pure ground glass nodules, (2) patients who underwent chemoradiotherapy, (3) patients who underwent CT-guided biopsy prior to the CT examination, (4) Preoperative CT examination was conducted within 2 weeks prior to surgery, and no lesion puncture was performed. For cases with multiple nodules, only the pulmonary nodules with definitive pathological results were included for subsequent analysis. The process of patient inclusion in this study is depicted in Figure 1.

This study collected clinical and pathological features such as age, gender, smoking history, semantic characteristics, pathological type, lymph node metastasis, neurovascular invasion, pleural invasion, and STAS of patients for analysis. Transparent Reporting of a Multivariable Prediction Model for Individual Prognosis or Diagnosis (TRIPOD) guidelines were followed in the study (19). The construction process of radiomics models is evaluated using the radiomics quality score (RQS) (20).

### Pathological diagnosis of STAS

After fixation in 10% formalin, the specimens were embedded in paraffin using conventional methods for subsequent pathological diagnosis by a pathologist. Following staining with hematoxylin and eosin, the tumor section was assessed under a multiheaded microscope. The diagnosis of STAS aligns with the 2015 World Health Organization pathological classification of lung tumors (21). STAS infiltrates the alveoli of the peripheral lungs in the form of clusters of micropapillary, solid nests, or single tumor cells.

### CT acquisition, ROI segmentation, and semantic characteristics

**Figure 2:**
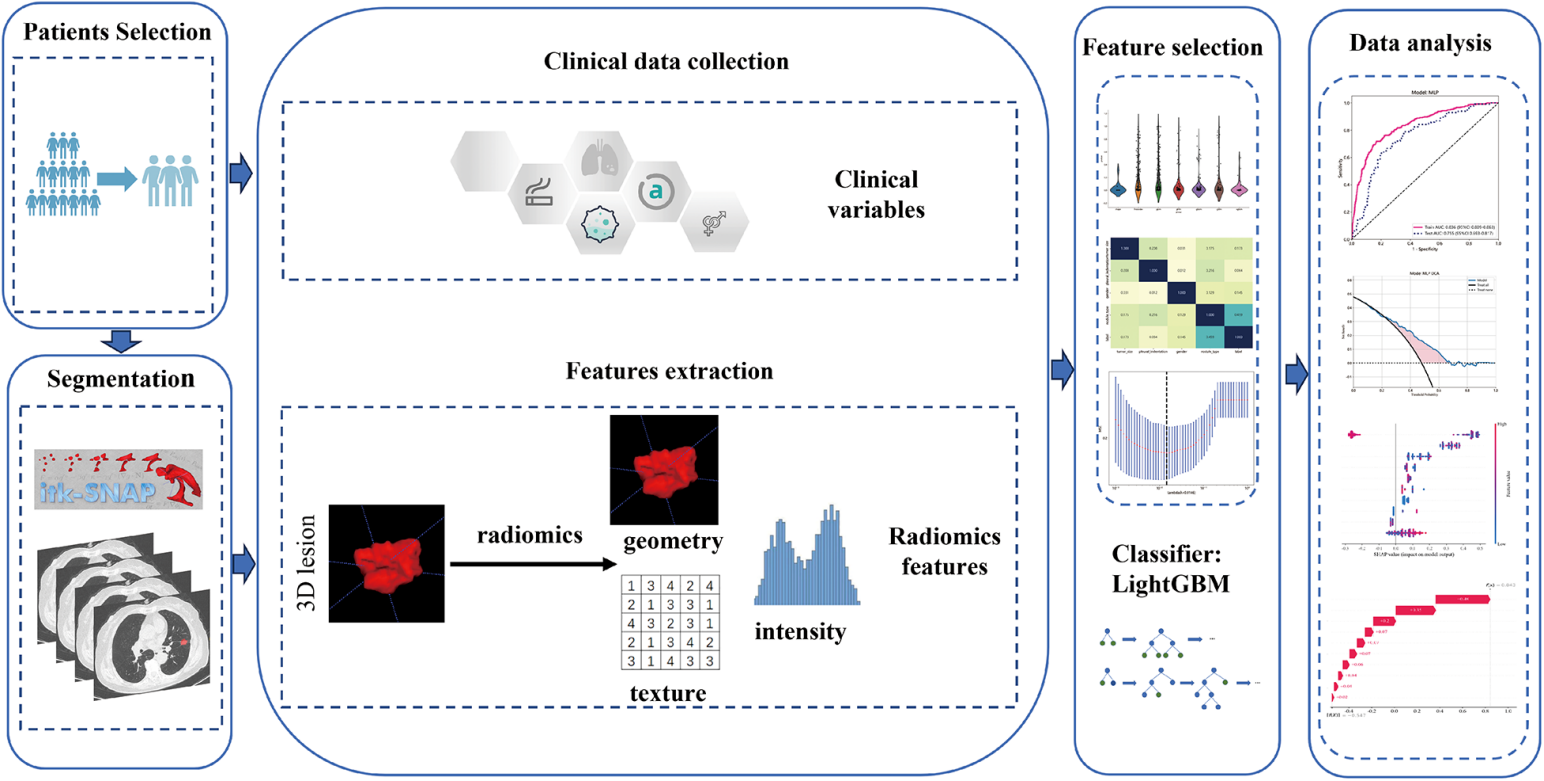
Workflow of radiomic analysis in this study.

The study utilized CT scans with a layer thickness ranging from 0.625 to 1.25 mm, without the administration of contrast medium. The bone reconstruction algorithm was employed for reconstruction purposes. Supplementary S1 contains detailed information regarding the acquisition and reconstruction parameters. The chest CT images underwent retrospective analysis by CT experts, possessing 5 and 10 years of experience, using a window width of 1,600 HU and a window level of −600 HU. This analysis aimed to determine semantic characteristics. The experts performed manual layer-by-layer segmentation of the tumor outline using ITK-SNAP (version 3.8.0, available at http://www.itksnap.org/). Any differences in opinion among the experts were resolved through discussion to achieve a consensus. Subsequently, the senior radiologist assessed the quality of the region of interest (ROI) and made necessary adjustments after the primary radiologist completed tumor lesion segmentation. To increase the robustness, 50 cases were randomly selected to estimate the intraclass correlation coefficients (ICCs), with a value of ≥0.75 indicating robustness. Figure 2 provides an illustration of the overall research design.

### Radiomics features extraction, selection and models construction

The “PyRadiomics” package in Python software was utilized for extracting tumor ROI features. This encompassed the extraction of shape features, grayscale features, wavelet transform features, and texture features. Supplementary S2 displays the classification and quantity of extracted radiomics features.

Following feature extraction, all radiomics features were standardized using the *z*-score for subsequent analysis. Moreover, we performed a statistical test employing the Mann-Whitney *U*-test to assess the significance of the features, selecting only those with a *p*-value < 0.05 for further consideration. We calculated the Spearman correlation coefficient to assess the correlation between features with high repeatability. If the correlation coefficient between two features exceeds 0.9, we keep only one feature to prevent redundancy. Feature selection involves utilizing the Least Absolute Shrinkage and Selection Operator (LASSO) to reduce feature dimensionality. The final radiomics features were inputted into the machine learning model, LightGBM.

The univariable and multivariable regression analysis was employed to analyze clinical and semantic features, and the variables ultimately included in the model demonstrated significant associations with the STAS status. The LightGBM was used for clinical model construction. To enhance the predictive ability, a comprehensive model was constructed by integrating radiomics signature with clinical signature. To evaluate the stability of the constructed models, we validated both the radiomics model and the clinical model across multiple cohorts.

### Model explanation and visualization

SHAP is a method for interpreting prediction results based on Shapley value theory, which decomposes the prediction results into the influence of each feature, providing global and local interpretability for the model. The core idea of SHAP is to allocate the contribution of feature values to different features, calculate the Shapley value of each feature, and multiply it with the feature value to obtain the contribution of that feature to the prediction result. SHAP can be used in machine learning models to generate visualized and quantitative interpretation results, helping doctors explain the decision-making process of the model. This study uses SHAP to interpret and visualize the LightGBM model.

### Performance evaluation and statistical analysis

In order to measure the accuracy of the models, receiver operating characteristic curve (ROC) were plotted to determine their diagnostic performance. Calibration curves were employed to evaluate the calibration performance of the models, and their calibration ability was further examined using the Hosmer-Lemeshow test. Additionally, the clinical utility of the predictive models was assessed through decision curve analysis (DCA). The area under the receiver operating characteristic curve (AUC), negative predictive value (NPV), positive predictive value (PPV), specificity, and sensitivity, were employed to compare the diagnostic performances of the three models in different cohorts.

The statistical analysis was conducted using SPSS and Python. The quantitative data obtained from the clinical and imaging features of the patients underwent normality tests using the Kolmogorov-Smirnov test and homogeneity of variance tests using Levene's test. Independent sample *T*-tests were utilized for data that met the assumptions of normality and homogeneity of variance. In cases where the assumptions were not met, Mann-Whitney *U*-tests were employed. For classification data, chi-square tests or Fisher tests were applied. Multivariate logistic regression analysis is employed to incorporate clinical variables into the construction of clinical models. The Spearman correlation coefficient to assess the correlation between features. The statistical significance of the results was assessed using a two-tailed *p*-value threshold of less than 0.05.

## Results

### Patient characteristics

The study included 1,325 patients, 1,168 from hospital 1, 93 from hospital 2, and 64 from hospital 3. Within the group of patients from hospital 1, the study divided them into two cohorts: a training cohort (*n* = 930) and a testing cohort (*n* = 238). External validation 1 involved 93 patients from hospital 2, while validation 2 encompassed 64 patients from hospital 3. Clinical and semantic features of the patients are summarized in Table 1. Within the training group, consisting of 930 patients, a division was made based on the presence or absence of STAS. Statistical analysis was conducted to evaluate the clinicopathologic characteristics, as presented in Table 2. Logistic regression analyses demonstrated that gender, nodule type, tumor size, and pleural indentation independently predicted the presence of STAS, as indicated in Table 3.

**Table 1:** The distribution of clinical characteristics and semantic features of different cohorts.

Characteristics	Total (*n* = 1,325)	Hospital 1		Hospital 2	Hospital 3
		Training (*n* = 930)	Testing (*n* = 238)	Validation 1 (*n* = 93)	Validation 2 (*n* = 64)
Age (years), mean ± SD	59.341 ± 9.678	58.843 ± 9.816	60.459 ± 9.431	60.412 ± 8.611	61.034 ± 9.565
Gender, *n* (%)					
Female	760 (57.4)	547 (58.8)	116 (48.7)	56 (60.2)	41 (64.1)
Male	565 (42.6)	383 (41.2)	122 (51.3)	37 (39.8)	23 (35.9)
Smoking, *n* (%)					
Yes	295 (22.3)	193 (20.8)	68 (28.6)	21 (22.6)	13 (20.3)
No	1,030 (77.7)	737 (79.2)	170 (71.4)	72 (77.4)	51 (79.7)
Tumor size (mm), mean ± SD	17.793 ± 5.532	17.375 ± 5.367	19.364 ± 5.899	18.602 ± 5.535	17.368 ± 5.417
Nodule type, *n* (%)					
PSN	780 (58.9)	567 (61.0)	144 (60.5)	40 (43.0)	29 (45.3)
Solid	545 (41.1)	343 (39.0)	96 (39.5)	53 (57.0)	35 (54.7)
Spicule, *n* (%)					
Yes	354 (26.7)	210 (22.6)	72 (30.3)	49 (52.7)	23 (35.9)
No	971 (73.3)	720 (77.4)	166 (69.7)	44 (47.3)	41 (64.1)
Lobulation, *n* (%)					
Yes	856 (64.6)	632 (68.0)	125 (52.5)	49 (52.7)	50 (78.1)
No	469 (35.4)	298 (32.0)	113 (47.5)	44 (47.3)	14 (21.9)
Pleural indentation, *n* (%)					
Yes	354 (26.7)	210 (22.6)	72 (30.3)	49 (52.7)	23 (35.9)
No	971 (73.3)	720 (77.4)	166 (69.7)	44 (47.3)	41 (64.1)
Bubble-like sign, *n* (%)					
Yes	141 (10.6)	92 (9.9)	29 (12.2)	15 (16.1)	5 (7.8)
No	1,184 (89.4)	838 (90.1)	209 (87.8)	78 (83.9)	59 (92.2)
STAS, *n* (%)					
Yes	510 (38.5)	351 (37.7)	114 (47.9)	34 (36.6)	11 (17.2)
No	815 (61.5)	579 (62.3)	124 (52.1)	59 (63.4)	53 (82.8)

SD, standard deviation; PSN, part-solid nodule; STAS, spread through air space.

**Table 2:** Baseline clinicopathological characteristics of lung adenocarcinoma patients in training cohort.

Variable	Total (*n* = 930)	STAS (+) (*n* = 351)	STAS (−) (*n* = 579)	*p*-value
Age (years), mean ± SD	58.843 ± 9.816	58.758 ± 9.909	58.881 ± 9.767	0.854
Gender, *n* (%)				<0.001*
Female	547 (58.8)	178 (50.7)	369 (63.7)	
Male	383 (41.2)	173 (49.3)	210 (36.3)	
Smoking, *n* (%)				0.001*
Yes	193 (20.8)	92 (26.2)	101 (17.4)	
No	737 (79.2)	259 (73.8)	478 (82.6)	
Tumor size (mm), mean ± SD	17.372 ± 5.367	18.473 ± 5.399	16.654 ± 5.234	<0.001*
Nodule type, *n* (%)				<0.001*
PSN	567 (61.0)	109 (31.1)	458 (79.1)	
Solid	363 (39.0)	242 (68.9)	121 (20.9)	
Spicule, *n* (%)				<0.001*
Yes	223 (24.0)	136 (38.7)	87 (15.0)	
No	707 (76.0)	215 (61.3)	492 (85.0)	
Lobulation, *n* (%)				<0.001*
Yes	632 (68.0)	283 (80.6)	349 (60.3)	
No	298 (32.0)	68 (19.4)	230 (39.7)	
Pleural indentation, *n* (%)				0.275
Yes	210 (22.6)	86 (24.5)	124 (21.4)	
No	720 (77.4)	265 (75.5)	455 (78.6)	
Bubble-like sign, *n* (%)				0.458
Yes	92 (9.9)	38 (10.8)	54 (9.3)	
No	838 (90.1)	313 (89.2)	525 (90.7)	
Histologic subtype, *n* (%)				<0.001*
AIS	7 (0.8)	0 (0.0)	7 (1.2)	
MIA	84 (9.0)	0 (0.0)	84 (14.5)	
IA	839 (90.2)	351 (100.0)	488 (84.3)	
Micropapillary, *n* (%)				<0.001*
Yes	269 (28.9)	203 (57.8)	66 (11.4)	
No	661 (71.1)	148 (42.2)	513 (88.6)	
Solid, *n* (%)				<0.001*
Yes	185 (19.9)	128 (36.5)	57 (9.8)	
No	745 (80.1)	223 (63.5)	522 (90.2)	
Lymph node metastasis, *n* (%)				<0.001*
Yes	94 (10.1)	82 (23.4)	12 (2.1)	
No	836 (89.9)	269 (76.6)	567 (97.9)	
Lymphovascular invasion, *n* (%)				<0.001*
Yes	77 (8.3)	66 (18.8)	11 (1.9)	
No	853 (91.7)	285 (81.2)	568 (98.1)	
Visceral pleural invasion, *n* (%)				<0.001*
Yes	93 (10.0)	69 (19.7)	24 (4.1)	
No	837 (90.0)	282 (80.3)	555 (95.9)	
Perineural invasion, *n* (%)				0.146
Yes	7 (0.8)	5 (1.4)	2 (0.3)	
No	923 (99.2)	346 (98.6)	577 (99.7)	

PSN, part-solid nodule; STAS, spread through air space; AIS, adenocarcinoma *in situ*; MIA, minimally invasive adenocarcinoma; IA, invasive adenocarcinoma.

* Represents *p* < 0.05.

**Table 3:** Univariate and multivariable logistic regression analyses for selecting clinical features of model development.

Variable	Univariate analysis		Multivariate analysis	
	OR (95% CI)	*p*-value	OR (95% CI)	*p*-value
Gender	1.154 (1.101, 1.209)	<0.001*	1.077 (1.023, 1.133)	0.017*
Age	0.999 (0.997, 1.002)	0.550		
Smoking	1.143 (1.080, 1.209)	<0.001*	1.021 (0.961, 1.084)	0.576
Tumor size	1.016 (1.011, 1.020)	<0.001*	1.009 (1.005, 1.013)	<0.001*
Nodule type	1.585 (1.517, 1.654)	<0.001*	1.529 (1.449, 1.613)	<0.001*
Pleural indentation	1.076 (1.019, 1.138)	0.028*	0.939 (0.891, 0.988)	0.044*
Lobulation	1.235 (1.177, 1.297)	<0.001*	1.030 (0.981, 1.080)	0.321
Spicule	1.378 (1.306, 1.454)	<0.001*	1.019 (0.958, 1.084)	0.619
Bubble-like sign	1.016 (0.938, 1.100)	0.745		

OR, odds ratio; CI, confidence interval.

* Represents *p* < 0.05.

### Radiomics features selection and radiomics model construction

**Figure 3:**
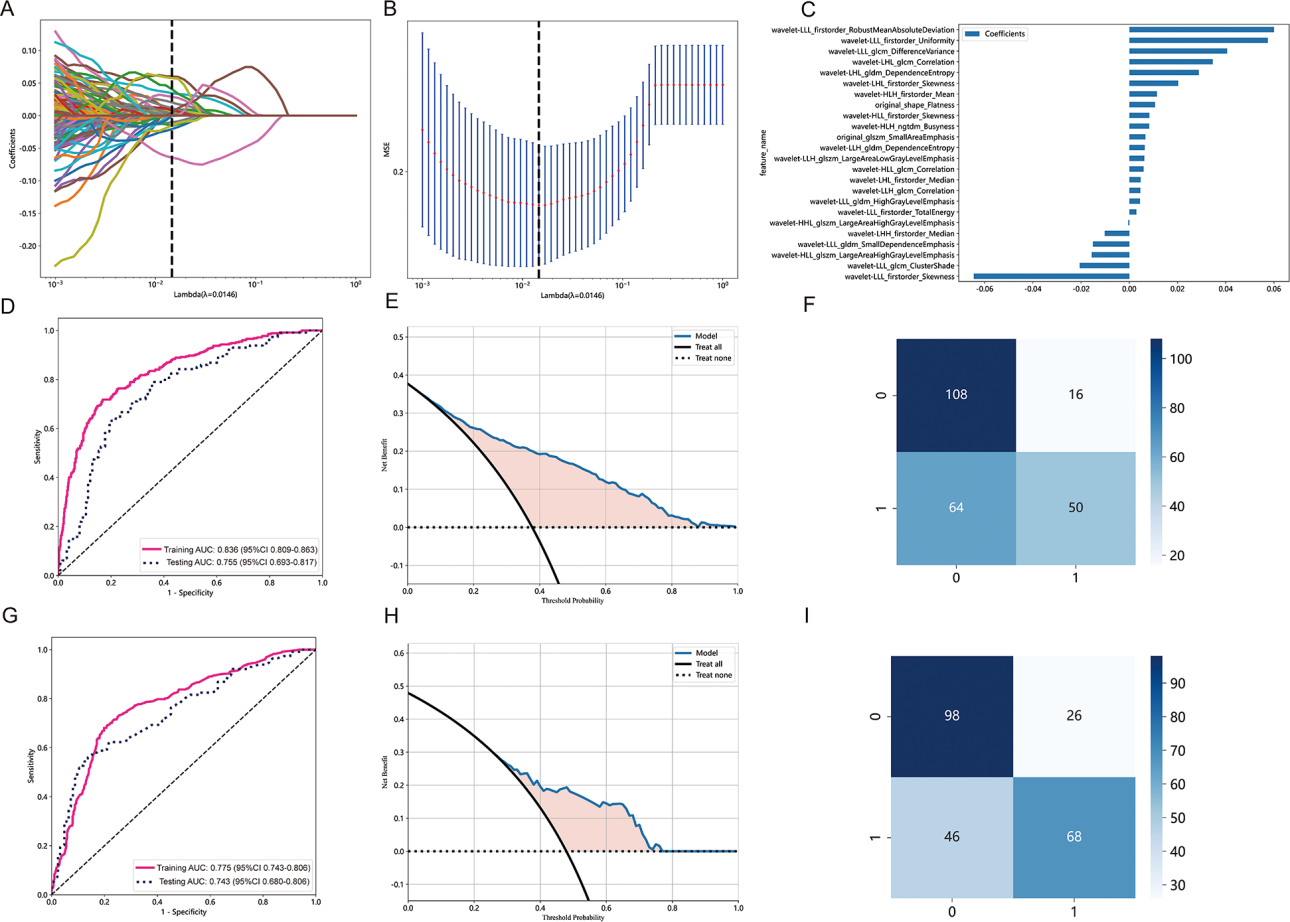
Radiomics feature selection based on LASSO algorithm and performance of the radiomics signature model. **(A)** LASSO coefficient profiles of the features. Different color line shows corresponding coefficient of each feature. **(B)** Tuning parameter (*λ*) selection in LASSO model. **(C)** Selected features weight coefficients. **(D)** The ROC curves of the radiomics signature model in the training and validation cohorts. **(E)** DCA for radiomics signature model. **(F)** Confusion matrix for radiomics signature model. **(G)** The ROC curves of the clinical model in the training cohort and testing cohort. **(H)** DCA for the clinical model. **(I)** Confusion matrix forthe clinical model.

A total of 833 radiomics features were extracted from the tumor ROI using PyRadiomics. 721 features were determined to be reliable after assessing their reproducibility using ICCs. Performing the Mann-Whitney *U* test resulted in the identification of 599 radiomic features. Subsequent analysis using the Spearman rank correlation test reduced the number of features to 196. By using the LASSO algorithm to reduce the dimensionality of features, 24 non zero coefficient radiomics features were ultimately selected. Figure 3A–C present the radiomics features with non-zero coefficients, as determined through LASSO penalized regression analysis. The performance of the radiomics model is depicted in Figure 3D, showing AUCs of 0.836 (95% CI: 0.809–0.863) and 0.755 (95% CI: 0.693–0.817) in the training cohort and the testing cohort. The corresponding DCA plot, displayed in Figure 3E, illustrated that the model consistently offers greater benefits in the majority of scenarios. Furthermore, Figure 3F showcased the confusion matrix for the radiomics model in the testing cohort, providing details such as an accuracy of 0.714, specificity of 0.645, sensitivity of 0.789, NPV of 0.769, and PPV of 0.672.

### Development of clinical model and combined model

Multivariable logistic regression analyses demonstrated that gender, nodule type, tumor size, and pleural indentation independently predicted the presence of STAS. These four clinical semantic features were used to construct a clinical model integrated into the LightGBM algorithm.

Figure 3G displays the performance of the clinical model, with AUCs of 0.775 (95% CI: 0.743–0.806) in the training cohort and 0.743 (95% CI: 0.680–0.806) in the testing cohort. Figure 3H showcases the predictive capability of the models in forecasting STAS, emphasizing their net benefit. Figure 3I presents the confusion matrix for the clinical model in the testing cohort, revealing an accuracy of 0.718, specificity of 0.847, sensitivity of 0.579, NPV of 0.686, and PPV of 0.776. Additionally, the combined model achieved AUCs of 0.837 (95% CI: 0.810–0.863) in the training cohort and 0.768 (95% CI: 0.707–0.829) in the testing cohort.

### Comparison of clinical model, radiomics model, and combined model

**Figure 4:**
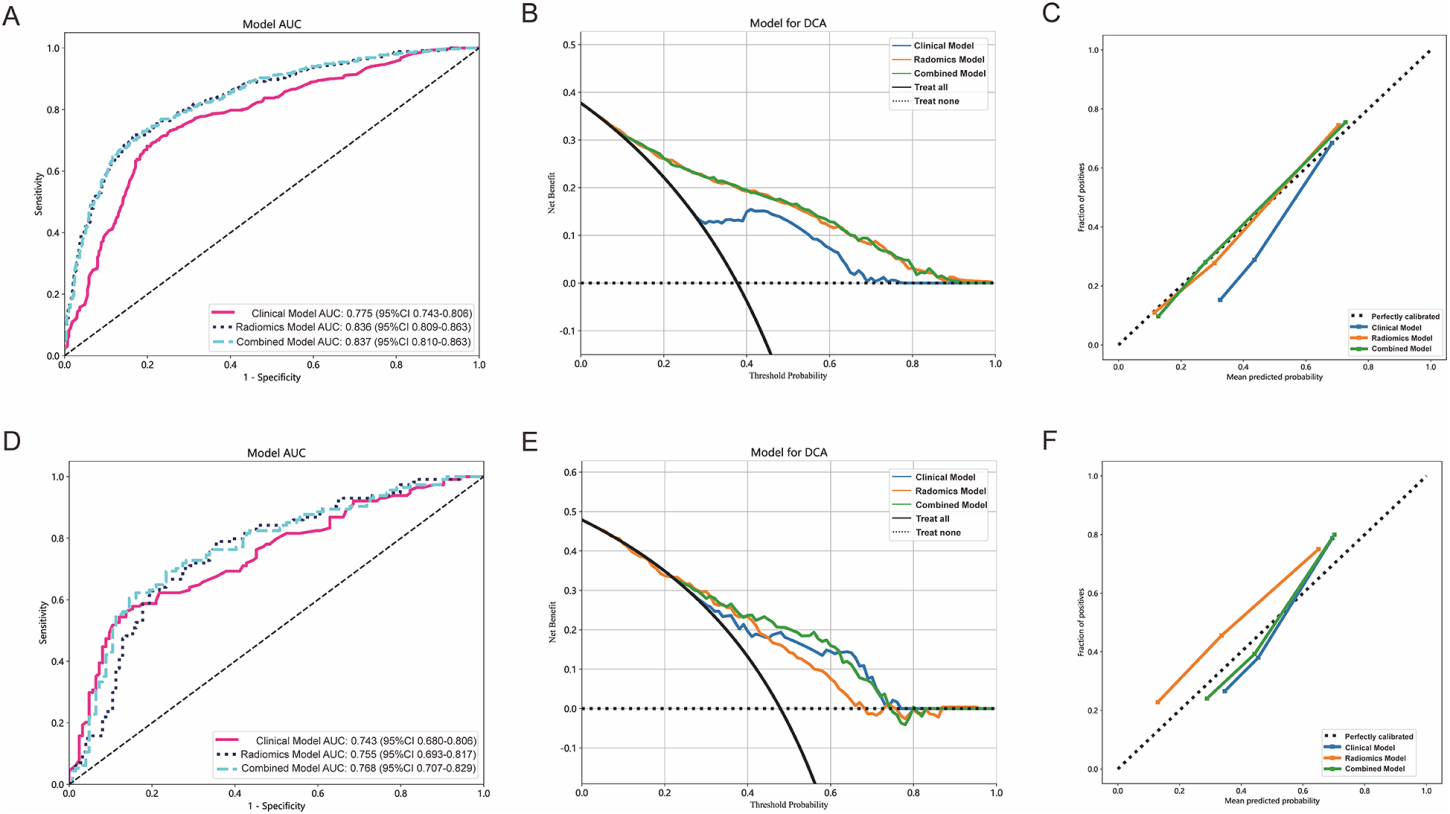
The performance of clinical model, radiomics model and combined model in the training and testing cohorts. The AUC, DCA and Calibration curves of clinical model, radiomics model, and combined model in the training cohort **(A–C)** and the testing cohort **(D–F)**.

In both the training and testing cohorts, the clinical model, radiomics model, and combined model were compared. The clinical model achieved an AUC of 0.775 in the training cohort and 0.743 in the testing cohort, as depicted in Figure 4A,D, respectively. Similarly, the radiomics model achieved an AUC of 0.836 in the training cohort and 0.755 in the testing cohort. The combined model, which integrated radiomics features, demonstrated an AUC of 0.837 in the training cohort and 0.768 in the testing cohort.

Figure 4B,E present the DCA of the three models, indicating that the combined model provided a net benefit in predicting STAS. Calibration curves in Figure 4C,F demonstrated agreement between the predicted and observed STAS in both cohorts. The Hosmer–Lemeshow *p*-values for the clinical model, radiomics model, and combined model were 0.421, 0.738, and 0.704, respectively. The DeLong test results indicated that the combined model outperformed the clinical model (*p* < 0.05), while no statistically significant differences were observed between the combined model and the radiomics model.

### Models validation and performance evaluation

**Figure 5:**
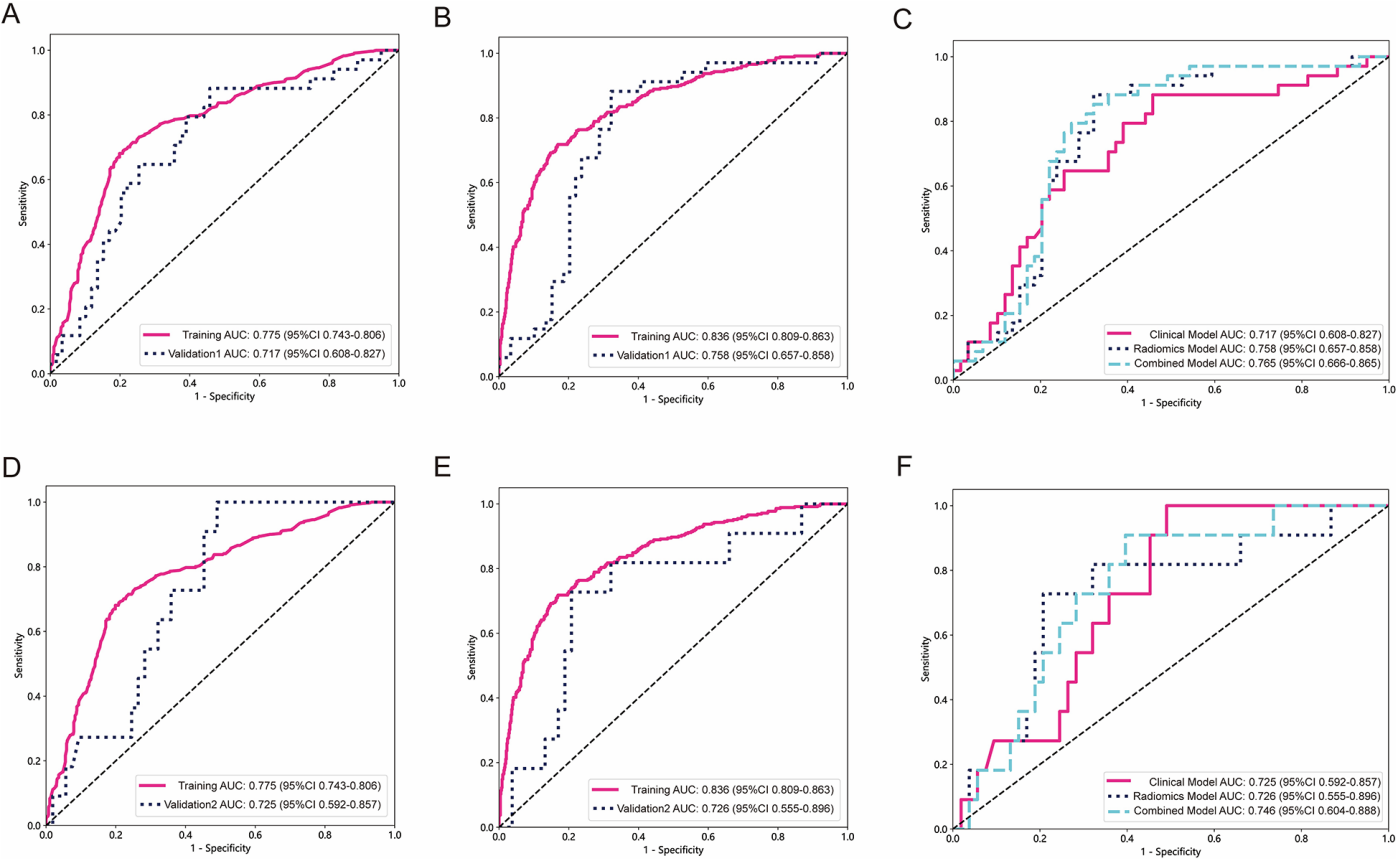
The performance of models in external validation. The ROC of the clinical model in validation 1 cohort and validation 2 cohort **(A,D)**. The ROC of the radiomics signature model in validation 1 cohort and validation 2 cohort **(B,E)**. The comparison of clinical model, radiomics model, and combined model in validation 1 cohort and validation 2 cohort **(C,F)**.

The AUC of clinical model was 0.717 (Figure 5A) in the validation 1 cohort, whereas the radiomics model (Figure 5B) and the combined model (Figure 5C) achieved AUCs of 0.758 and 0.765, respectively. In the validation 2 cohort, The AUC of the clinical model (Figure 5D) was 0.725, while the radiomics model (Figure 5E) and the combined model (Figure 5F) achieved AUCs of 0.726 and 0.746, respectively. The performance of the clinical model, radiomics model, and combined model in the training cohort, testing cohort, validation 1 cohort, and validation 2 cohort is summarized in Table 4. This study received 18 RQS points and achieved a total score of 50%. According to Supplementary S4, this signature is classified as TRIPOD 3.

**Table 4:** The model performances in the training cohort, testing cohort, validation 1 cohort and validation 2 cohort.

	AUC	95% CI	Sensitivity	Specificity	PPV	NPV
Training cohort						
Clinical model	0.775	0.743–0.806	0.681	0.801	0.675	0.806
Radiomics model	0.836	0.809–0.863	0.718	0.831	0.720	0.829
Combined model	0.837	0.810–0.863	0.729	0.812	0.701	0.832
Testing cohort						
Clinical model	0.743	0.680–0.806	0.579	0.847	0.776	0.686
Radiomics model	0.755	0.693–0.817	0.789	0.645	0.672	0.769
Combined model	0.768	0.707–0.829	0.623	0.846	0.780	0.707
Validation 1 cohort						
Clinical model	0.717	0.608–0.827	0.882	0.542	0.526	0.889
Radiomics model	0.758	0.657–0.858	0.882	0.678	0.612	0.909
Combined model	0.765	0.666–0.865	0.853	0.678	0.604	0.889
Validation 2 cohort						
Clinical model	0.725	0.592–0.858	1.000	0.510	0.297	1.000
Radiomics model	0.726	0.555–0.896	0.727	0.808	0.421	0.933
Combined model	0.746	0.604–0.888	0.909	0.615	0.323	0.323

AUC, area under curve; CI, confidence interval; PPV, positive predictive value; NPV, negative predictive value.

### Explanation and visualization of radiomics model

**Figure 6:**
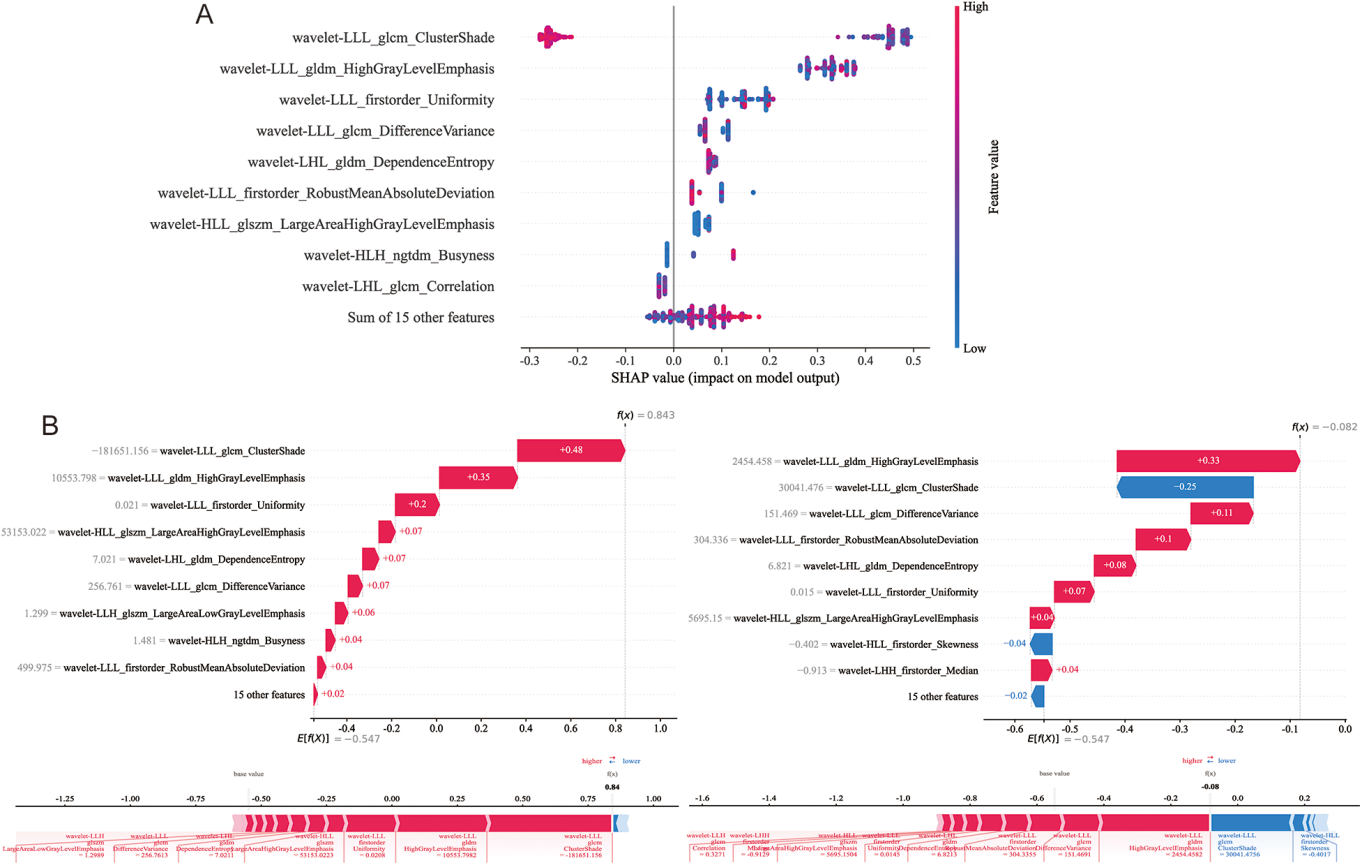
Model interpretability display and case application analysis. **(A)** Shapley summary diagram of the LightGBM model. **(B)** Application analysis for two patients with STAS (+) and STAS (−).

SHAP is used to visualize the global interpretation of each feature contribution in the LightGBM model. In Figure 6A, Arrange the importance of features from top to bottom, and the horizontal axis of the graph displays the SHAP values of the features, with each point representing a patient. The color of a point is determined by its feature value, with the redder the color, the higher the feature value, and the bluer the color, the lower the feature value. Wavelet-LLL_glcm_ClutureShde is considered the most important feature. As shown in Figure 6B, the waterfall visualization LightGBM model generated by SHAP is used to describe the decision-making process of whether two lung adenocarcinoma patients have STAS. Based on the contribution of each feature to the decision, all features are arranged in order, and the direction of their contribution is displayed by color. The score calculation starts from *E*[*f* (*x*)], and then the SHAP values are added together. Red indicates an increase in the probability of STAS, blue indicates a decrease in the probability of STAS, and ends with individual prediction.

## Discussion

This multicenter study focused on the development of a prediction biomarker for noninvasive preoperative detection of STAS in patients with clinical stage IA lung adenocarcinoma. Our model integrated clinical independent risk factors and radiomics features, demonstrating excellent predictive ability and reproducibility across diverse cohorts.

Lung cancer, one of the most prevalent malignancies (22), has seen an increased detection rate of early-stage cases thanks to CT screening of high-risk groups in recent years (23). Histologically, most early-stage lung cancers are confirmed as adenocarcinomas (24). Studies have established sub-lobar resection with selective lymph node dissection, as a viable alternative to the standard treatments of anatomical lobectomy and systematic lymph node dissection for early-stage lung cancer (25). However, sub-lobar resection can lead to high recurrence and metastasis rates, resulting in a poor prognosis for some patients (26). Advancements in pathological research have revealed STAS as another form of invasion or metastasis, in addition to lymph node metastasis, hematogenous metastasis, and local implantation metastasis (27). STAS status has shown associations with various pathologic characteristics. In our study, we observed a significant correlation between STAS and the micropapillary growth pattern, solid components, lymph node metastasis, and lymphovascular invasion, consistent with previous findings (28, 29). Previous studies have reported STAS incidence in lung adenocarcinoma ranging from 14.8% to 56.4%, identifying it as a risk factor for postoperative survival and recurrence (1, 6, 30). Among our cohort of 1,325 patients, pathologically confirmed STAS was present in 510 (38.5%) cases. Interestingly, our investigation revealed a higher prevalence of STAS in male smokers. The duration and quantity of tobacco consumption are closely associated with lung cancer development (31). The impact of smoking on pulmonary nodules' biological characteristics can result in increased invasiveness. Our research findings indicate that gender and smoking can serve as predictive factors for identifying STAS, as determined through univariate logistic regression analyses.

Previous studies have established that CT features can serve as predictive indicators of STAS, including nodule type, tumor size, spiculation, lobulation, and pleural indentation (29). Thoracic surgeons consider nodule type and tumor size crucial factors when choosing surgical strategies, as they have been identified as predictors for STAS in previous studies (12, 32). Our research findings confirm the independent predictive value of tumor size and nodule type for identifying STAS, as determined through univariate and multivariate logistic regression analyses. Regarding radiological features, our study found that spicule and lobulation were more common in the positive STAS group. Univariate analysis demonstrated their predictive value for STAS, although the multivariate analysis did not reach statistical significance. Although there was no statistically significant difference in pleural indentation between the STAS positive and STAS negative groups. Pleural indentation was identified as an independent predictive factor for STAS in both univariate and multivariate logistic regression analyses. Furthermore, we constructed a clinical prediction model by incorporating clinical characteristics with statistically significant differences in the multivariate logistic regression. The AUC values for the training, testing, external validation 1, and validation 2 cohorts were 0.775, 0.743, 0.717, and 0.725, respectively. These features, along with radiomics features, were used to develop and validate a combined model to discriminate STAS. In all datasets, the combined model exhibited a higher AUC compared to the radiomics model, while the radiomics model yielded a higher AUC than the clinical model. Statistical differences were observed between the combined model and clinical models, as well as between the radiomics model and clinical models. However, no statistical differences were found between the combined model and the radiomics model. These research findings suggest that the inclusion of clinical features in the combined model does not significantly enhance its performance, emphasizing the potential of radiomics features as valuable biomarkers for preoperative CT-based prediction of STAS.

Several studies have consistently shown that limited resection in stage IA lung adenocarcinoma patients with STAS leads to significantly lower rates of RFS and OS compared to lobectomy (3). Notably, STAS in stage IA lung cancer patients treated with lobectomy no longer poses a significant risk for recurrence and overall survival (33). Therefore, accurately predicting the presence of STAS is crucial for guiding surgical strategies in early-stage lung cancer. To date, several studies have focused on preoperative prediction of STAS. Previous research has explored the use of clinical factors and CT characteristics to predict STAS status. Ding et al. developed a nomogram prediction model using clinical features that achieved an AUC of 0.724 for diagnosing STAS (34). Intraoperative freezing has also been suggested as a diagnostic method for STAS, but it exhibits low sensitivity (11, 35, 36). Furthermore, research has described the development of a stepwise flowchart for decision-making on sublobar resection in early-stage lung cancer, based on preoperative PET-CT and frozen section analysis to estimate the extent of spread through air space. However, the AUC of GGO (2D) on CT was 0.70, and the AUC of PET-CT T/L ratio was 0.72 (37), which are lower than the prediction models constructed in our study. The precise preoperative assessment of STAS plays a crucial role in guiding surgeons towards appropriate surgical strategies. In this study, machine learning algorithms were employed to develop a CT radiomics model for the prediction of STAS. The AUCs of model were 0.758 and 0.726 in the external validation 1 and validation 2 datasets, respectively. These findings hold significant clinical application value as they can serve as a reference for formulating individualized diagnostic and treatment approaches for early-stage lung adenocarcinoma patients.

In machine learning and data science, the interpretability of models has always been a concern. Explainable Artificial Intelligence (XAI) enhances trust in models by increasing model transparency. The SHAP library is an important tool that provides visualization functionality by quantifying the contribution of features to prediction. The advantages of SHAP include strong interpretability, high accuracy, and applicability to various models and feature types. Our research visualizes the decision-making process of the LightGBM model through SHAP, which can help doctors better understand the prediction results of machine learning models, identify model weaknesses, and improve the model.

The present study had several limitations. Firstly, this study is retrospective in nature, which may introduce potential selection bias. Future research aims to validate the model's feasibility through prospective experiments. Secondly, the study exclusively focused on adenocarcinoma and did not encompass other tumor types. Our future plan involves extracting features from various pathological types of lung cancer to build models and evaluate the efficacy of radiomics in predicting STAS in those categories. Thirdly, controversies exist regarding the subjective nature of manually defining segmentation boundaries. Our future goal is to attain full automation through deep learning.

## Conclusion

The CT-based radiomics model demonstrated satisfactory diagnostic performance in predicting STAS in clinical stage IA lung adenocarcinoma. This approach exhibits potential as a non-invasive biomarker for preoperatively predicting STAS in clinical surgical decision making.

## Data Availability

The original contributions presented in the study are included in the article/Supplementary Material, further inquiries can be directed to the corresponding authors.
